# The effects of age at menarche and first sexual intercourse on reproductive and behavioural outcomes: A Mendelian randomization study

**DOI:** 10.1371/journal.pone.0234488

**Published:** 2020-06-15

**Authors:** Rebecca B. Lawn, Hannah M. Sallis, Robyn E. Wootton, Amy E. Taylor, Perline Demange, Abigail Fraser, Ian S. Penton-Voak, Marcus R. Munafò

**Affiliations:** 1 MRC Integrative Epidemiology Unit at the University of Bristol, Bristol, United Kingdom; 2 School of Psychological Science, University of Bristol, Bristol, United Kingdom; 3 Department of Population Health Sciences, Bristol Medical School, University of Bristol, Bristol, United Kingdom; 4 NIHR Biomedical Research Centre at the University Hospitals Bristol NHS Foundation Trust and the University of Bristol, Bristol, United Kingdom; 5 Département d’Etudes Cognitives, Ecole Normale Supérieure, Paris, France; University of Jyvaskyla, FINLAND

## Abstract

There is substantial variation in the timing of significant reproductive life events such as menarche and first sexual intercourse. Life history theory explains this variation as an adaptive response to an individual’s environment and it is important to examine how traits within life history strategies affect each other. Here we applied Mendelian randomization (MR) methods to investigate whether there is a causal effect of variation in age at menarche and age at first sexual intercourse (markers or results of exposure to early life adversity) on outcomes related to reproduction, education and risky behaviour in UK Biobank (N = 114 883–181 255). Our results suggest that earlier age at menarche affects some traits that characterize life history strategies including earlier age at first and last birth, decreased educational attainment, and decreased age at leaving education (for example, we found evidence for a 0.26 year decrease in age at first birth per year decrease in age at menarche, 95% confidence interval: -0.34 to -0.17; p < 0.001). We find no clear evidence of effects of age at menarche on other outcomes, such as risk taking behaviour. Age at first sexual intercourse was also related to many life history outcomes, although there was evidence of horizontal pleiotropy which violates an assumption of MR and we therefore cannot infer causality from this analysis. Taken together, these results highlight how MR can be applied to test predictions of life history theory and to better understand determinants of health and social behaviour.

## Introduction

Life history theory addresses how organisms differ in allocation of limited resources between growth and reproductive efforts, characterizing species into those on ‘fast’ or ‘slow’ life history strategies [1,2]. Life history theory can be considered a meta-theory and can therefore not be tested in its entirety but can generate testable predictions [3,4]. The literature on life history theory has become increasingly large and fragmented in recent years [4]. The most commonly tested prediction is the association between harsh early life environments and a fast life history strategy however it is also important to examine how traits within life history strategies affect each other. A ‘fast’ life history strategy is characterised by more effort directed towards reproduction such as earlier puberty and sexual activity, whereas a ‘slow’ life history strategy can be described by later maturity and proportionally greater investment in a smaller number of offspring [1,2]. For example, rabbits undergo rapid sexual development, short interbirth intervals and various other traits demonstrating short-term goals that characterize a fast life history strategy [5]. Conversely, elephants show delayed sexual development and long interbirth intervals and are considered to be on a slow life history strategy [5]. The shorter life expectancy of rabbits than elephants increases the adaptive benefits of taking a fast life history strategy [5].

Within-species variation in life history strategy has been proposed. For humans, life history theory has been applied to characterize individuals into those on relatively faster or slower strategies [5], with substantial variation between humans in the timing of significant reproductive life events such as age at menarche (the start of a woman’s sexual maturity and reproductive potential) and first sexual intercourse [6,7]. Life history theory explains this variation as an adaptive response to an individual’s developmental environment and adverse childhood experiences have been shown to associate with earlier age at menarche [8] and earlier age at first sexual intercourse [9–11]. Life history strategies consist of a suite of adaptations and whilst adopting a fast life history strategy evolved due to reproductive advantages in certain conditions, it may also have costs to an individual in modern environments. Such costs include those associated with teenage pregnancy and risky behaviours like violence, criminality, and substance abuse [1,12,13]. Therefore, as well as previous research into the causes of earlier age at menarche and sexual intercourse (such as early life adversity) [8–11], it is also important to examine how traits within life history strategies affect each other, especially as starting menarche and sexual intercourse is necessary for reproductive life history outcomes and age at first sexual intercourse may be modifiable via policy and environmental changes. In the present study, we examine how traits within life history strategies affect each other by examining the effects of these two reproductive traits (age at menarche and age at first sexual intercourse) on other reproductive and behavioural outcomes including age at first birth, age at last birth and educational attainment. Knowledge on the suite of possible adaptations following these two reproductive traits is important for research investigating the effects of reproductive timings on later life health outcomes and for insight into key predictors of educational attainment.

Standard analytical approaches applied to observational data have been used to examine life history strategies in humans as it is not easily possible to manipulate developmental environments or reproductive timings in experimental settings [11,14]. However, inferring causality in studies using such approaches is difficult, and likely to be affected by confounding [15]. For example, structural equation modelling has been proposed to investigate life history theory, however the potential for confounding is always a concern as these methods allow for the control of measured but not for unmeasured confounders [16,17]. Even though it is difficult to manipulate reproductive timings, particularly age at menarche but also age at first sexual intercourse, we can apply the method of Mendelian randomization (MR) to investigate causal relationships between these traits and outcomes of interest. MR is an increasingly popular method in epidemiology for strengthening causal inference when randomized controlled trials cannot be conducted and there is no possibility of manipulating risk factors [18].

**Fig 1:**
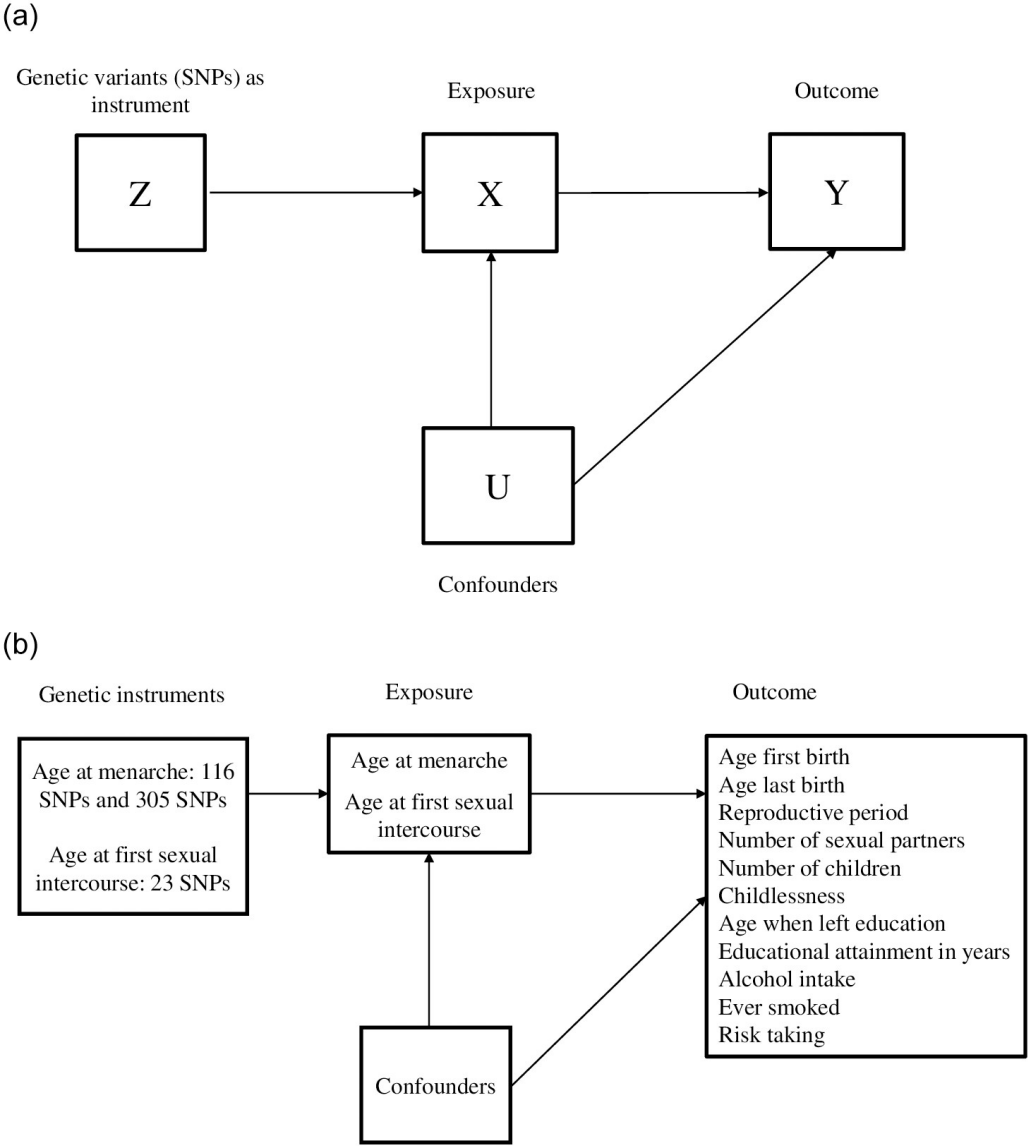
**A.** Diagram representing Mendelian randomization analysis. **b.** Diagram representing Mendelian randomization analyses in the present study.

MR employs an instrumental variable analysis framework, with the instrument specifically being genetic variants known as single nucleotide polymorphisms (SNPs)[15]. The genetic instrument (‘Z’) is used as a proxy for an environmental exposure of interest to investigate the effect of this exposure (‘X’) on an outcome (‘Y’) (see Fig 1a)[15,19,20]. Valid instrumental variables are defined by three main assumptions to allow for causal inference of results [19]. First, the instrument is robustly associated with the exposure (the relevance assumption). Genetic variants used as instrumental variables in MR are identified in genome-wide association studies (GWAS) to be significantly and independently associated with the exposure at a *p*-value less than 5×10^−8^. Second, the instrument is not associated with confounding factors (‘U’) (the independence assumption). By using independent genetic variants as instruments, MR exploits Mendel’s laws of segregation and independent assortment by which the inheritance of genetic variants is determined mostly independently of other genetic variants and the environment [15]. This independence has been demonstrated through pairwise correlations between nongenetic variables and genetic variables, with genetic variants showing little association with each other [21]. This highlights the advantages of using genetic variants as proxies of environmental exposures to overcome bias due to confounding to which non-genetic observational studies are prone [21]. Third, the instrument only affects the outcome through its effect on the exposure (the exclusion restriction assumption). This third assumption is violated in the presence of horizontal pleiotropy: when the genetic variant has an effect on the outcome through alternative pathways, instead or in addition to, through the exposure [22,23]. The presence of horizontal pleiotropy is investigated in analyses [22,23]. Additionally, since genotype is determined at conception, MR removes the risk of reverse causality [15,19]. If these assumptions are met, effects estimated using MR should be free from bias due to confounding [15] and the associations between ZX and ZY (i.e., SNP-exposure and SNP-outcome) can be used to estimate the causal effect of the exposure (‘X’) on outcome (‘Y’) (see Fig 1a and 1b) [24]. This causal effect is obtained through calculating a Wald ratio where the SNP-outcome estimate is divided by SNP-exposure estimate (ZY÷ZX) and forms the basis for all MR methods used here. These SNP-exposure and SNP-outcome associations should be estimated from distinct non-overlapping samples of participants [24].

One example of using MR to overcome biases when manipulation of the exposure is not practical is the study of alcohol consumption effects on blood pressure and, ultimately, cardiovascular disease (previously described in [15] and [18]). Individuals who consume more alcohol may differ from individuals who consume less alcohol for other cardiovascular risk factors, such as by smoking more heavily. This could therefore introduce spurious associations due to bias from confounding by smoking heaviness. Using SNPs associated with metabolite responses to alcohol consumption is akin to randomizing individuals into higher or lower drinking conditions [24,25] and MR can therefore be used to estimate a causal effect of alcohol consumption. For more information on interpreting MR studies, see Davies et al. [18].

Here we apply the logic of MR to estimate the causal effects of one reproductive trait on reproductive and behavioural outcomes within a life history framework. Using instruments for age at menarche (and a separate instrument for age at first sexual intercourse), we independently investigate the effects of age at menarche and age at first sexual intercourse on several evolutionary relevant outcomes (see Fig 1b). MR has been used previously to investigate age at menarche with many later life health outcomes (e.g., Sequeira et al. [26]) however in this study we aim to provide stronger causal inference for age at menarche and evolutionary relevant outcomes. Whereas smoking heaviness may have confounded relationships in the above example (see Fig 1a), socioeconomic status may be one factor with similar effects in observational studies of age at menarche and age at first sexual intercourse and these outcomes.

Earlier age at menarche and age at first sexual intercourse can be viewed as directing effort towards reproductive goals as part of a fast life history strategy. In line with this, we predict earlier age at menarche and age at first sexual intercourse to be causal components of a suite of adaptations where the future is discounted relative to the present and effort is directed towards short-term reproductive goals and increased risky behaviour [1,27]. For example, short-term reproductive goals may include earlier age at first birth, earlier age at last birth, a shorter reproductive period, increased number of sexual partners and number of children, and less likelihood of being childless. Increased risky behaviour could manifest as increased likelihood of smoking and alcohol consumption in the modern day. On the other hand, investing in education, despite being evolutionarily novel, can be seen as a slow life history trait with delayed benefits [27].

A previous study [27] that included a sub-sample of participants from UK Biobank showed a causal effect of earlier age at menarche on earlier age at first birth, earlier age at last birth, earlier age at leaving education, increased alcohol intake, lower likelihood of being childless, greater number of children (in combined sexes) and decreased likelihood of remaining in education after 16 years. Additionally, earlier age at first sexual intercourse was causally related to earlier age at first birth, a greater number of children, increased likelihood of being an ever smoker, and decreased likelihood of attaining a degree. These findings suggest causal relationships between traits that characterize a life history strategy and support evolutionary explanations of variation in age at menarche and first sexual intercourse. We extend this work by using the full release of UK Biobank data (N = 114 883–181 255) and a suite of novel methods to more robustly test for horizontal pleiotropy, which would violate one of the key assumptions of MR.

## Methods

### Exposure data

For our age at menarche instruments, we used independent SNPs associated with age at menarche (*p*<5×10^−8^) from two GWAS separately [28,29]. The first identified 123 SNPs [28] and explained approximately 3% of the observed variance in age at menarche (N = 182 416). The second identified 389 SNPs which explained approximately 7% of the variance (N = 329 345) [29]. After excluding certain SNPs for methodological reasons (see S1 Text), we were left with 116 and 305 SNPs as two instruments for age at menarche. Mean differences and standard errors (SE) for these SNPs and age at menarche associations in the GWAS discovery samples were recorded for each instrument and this became our exposure data for age at menarche (see S1 and S2 Tables).

For our instrument of age at first sexual intercourse, we used independent SNPs associated with age at first sexual intercourse (*p*<5×10^−8^) [27] in both males and females. We recorded these GWAS associations, as done so for age at menarche, to be used as our exposure data for age at first sexual intercourse (see S3 Table). We used effect estimates identified in the pooled sex GWAS to increase statistical power. Of the 33 SNPs for the instrument of age at first sexual intercourse, there were 23 SNPs available in UK Biobank (see S1 Text).

### Outcome data

The exposure associated SNPs described above were extracted from UK Biobank to derive SNP-outcome associations for each outcome. Extraction was done using PLINK (v2.00) and best guess algorithms for determining alleles.

#### Sample

UK Biobank is a population-based health research resource consisting of approximately 500 000 people, aged between 38 years and 73 years, who were recruited between the years 2006 and 2010 from across the UK [30]. Particularly focused on identifying determinants of human diseases in middle-aged and older individuals, participants provided a range of information (including as demographics, health status, lifestyle measures, cognitive testing, personality self-report, and physical and mental health measures) via questionnaires and interviews (data available at www.ukbiobank.ac.uk). A full description of the study design, participants and quality control (QC) methods have been described in detail previously [31]. UK Biobank received ethics approval from the Research Ethics Committee (REC reference for UK Biobank is 11/NW/0382). Genotyping information and quality checks in UK Biobank are described elsewhere [32].

#### Outcome measures

Our outcome measures were: age at first birth, age at last birth, reproductive period, number of children, childlessness, ever smoked, educational attainment in years, age when left education, alcohol intake, risk taking and number of sexual partners for those that indicated they had had sex. These measures were derived similarly to previous research [27,33]. We re-coded data as missing if age at first sexual intercourse was younger than age at menarche; if age at leaving education was answered as having never attended school; at the 99.99^th^ percentile for number of children; at the 99.99^th^ percentile for number of sexual partners. Reproductive period was derived as the difference between age at last birth and age at first birth for those that had more than one child. To account for non-normal data, we included binary measures of childlessness (childlessness coded as 1). We also included a measure for ever smoked (coded as 1 if participants had ever smoked in questions ‘Do you smoke tobacco now?’ or ‘In the past, how often have you smoked tobacco?’). Alcohol intake was a categorical variable indicating ‘never’ (coded as 6), ‘special occasions only’, ‘one to three times a month’, ‘once or twice a week’, three to four times a week’ and ‘daily or almost daily’ (coded as 1). Risk taking was measured as ‘yes’ (coded as 1) or ‘no’ responses to ‘Would you describe yourself as someone who takes risks?’. Only females were used for all outcome data.

### Data analysis

Data were harmonized to ensure that the effect of the SNP on the exposure and the SNP on the outcome corresponded to the same allele. The age increasing allele was used in order to conduct MR analyses and results were then reversed to report the effect of earlier age at menarche and first sexual intercourse. To derive the SNP-outcome associations for our outcome data, regressions were conducted in R adjusting for birth year and the top 10 genetic principal components. In sensitivity analysis, we additionally adjusted SNP-outcome associations for genotype array.

We used the 116 SNPs for age at menarche [28] in our main analysis as this GWAS did not include any UK Biobank data. For the 305 SNP instrument which includes some individuals from the UK Biobank [29], we calculated SNP-outcome associations and conducted analysis using outcome data from a UK Biobank sub-sample that did not overlap with the age at menarche GWAS. However, allocation into these sub-samples is related to smoking status [34] and division is therefore similar to stratifying on smoking. As smoking may be a collider in our analysis, this stratification could introduce bias. We therefore also derived SNP-outcome estimates and conducted analysis for the 305 SNP age at menarche instrument using the full UK Biobank sample, which will suffer from bias towards the observational estimate due to sample overlap with the GWAS of the exposure [35]. It is also not possible to assess the suitability of one MR method (MR-Egger, described below) with sample overlap as the suitability value (the I^2^_GX_ value) cannot be reliably measured. As the age at first sexual intercourse GWAS [27] was conducted solely in a sub-sample of UK Biobank participants, we conducted a fixed-effects meta-analysis of the SNP-outcome estimates in the full UK Biobank sample in addition to MR analysis in the non-overlapping sub-sample of UK Biobank. This was only conducted for age at first sexual intercourse and not also for age at menarche. This fixed-effects meta-analysis is equivalent to performing an unweighted allele score analysis [36] and suffers from less bias than a weighted analysis with overlapping samples. The units for this fixed effect meta-analysis therefore differ to the other MR methods as it is per increase in the number of effect alleles.

Exposure and outcome data (i.e., SNP-exposure and SNP-outcome associations) were combined using multiple MR approaches (inverse variance weighted, weighted median, weighted mode-based estimator (MBE) and MR-Egger methods). These methods are all extensions of the Wald ratio (defined above), to be used with multiple SNPs as instruments for the exposure. They first use the Wald ratio to estimate the causal effect per SNP before conducting a meta-analysis for the causal effect of an exposure on outcome across SNP instruments (see Bowden & Holmes [37] for a recent review of meta-analyses for MR). Each method uses a varying number of the SNPs as instruments due to the different assumptions that each relies on. With these methods each relying on different assumptions regarding horizontal pleiotropy, a consistent effect across all methods increases our confidence in results, although some methods suffer from reduced statistical power [23]. An inverse variance weighted approach is analogous to a weighted regression of SNP-outcome coefficients on SNP-exposure coefficients with the intercept constrained to zero [38,39], and further includes all SNPs by assuming all are valid instruments (i.e. meet the relevance, independence and exclusion restriction assumptions defined above) or allows pleiotropy to be balanced across instruments when using the random effects model [39]. The weighted median estimate is obtained by first calculating the Wald ratio for each SNP and then taking the estimate with the median inverse variance weight. The weighted median method estimates a causal effect if at least 50% of the data for analysis is from variants that are valid instruments (i.e., meet the relevance, independence and exclusion restriction assumptions defined above) [19,40]. The weighted MBE finds the largest cluster of Wald ratios for a meta-analysis and provides a causal estimate when the largest number of similar individual-instrument estimates come from valid instruments, even if the majority are invalid [41]. A tuning parameter of 0.5 was set for MBE analysis. We further conducted MR-Egger regression which allows all variants to have pleiotropic effects if they are independent to the variants’ effects on the exposure [23]. MR-Egger does not constrain the intercept to zero and the intercept term therefore estimates overall horizontal pleiotropy [23]. In addition to these analyses, we conducted Radial MR and a leave one out analysis for age at first sexual intercourse which helps to identify outlier SNPs [42]. For binary outcomes, all MR results were transformed to odds ratios by exponentiating them.

We calculated Cochran’s Q for these inverse variance weighted analyses to test if effects differ across variants [18]. We further calculated the I^2^_GX_ to assess the suitability of MR-Egger where above 0.9 is desired [43], and mean F statistics which indicate the strength of the instrument. For age at first sexual intercourse, the unweighted I^2^_GX_ was low and we therefore performed a SIMEX adjustment with unweighted analysis.

Age at menarche analysis using the 116 SNP instrument was repeated after removing SNPs associated with body mass index at *p* < 5 × 10^−8^ [26,36,44]. This resulted in 9 SNPs being removed (rs10938397, rs12446632, rs2947411, rs3101336, rs543874, rs7103411, rs7138803, rs7514705, rs8050136).

## Results

Mean age in our sample was 57 years (standard deviation [SD] = 7.91). Mean age at menarche and first sexual intercourse were 13 years (SD = 1.60) and 19 years (SD = 3.44), respectively. Further sample characteristics are given in Table 1. Further details of the instruments are provided in S4 Table.

**Table 1:** Population characteristics of UK Biobank sample used as outcome data.

	Total N	Mean (SD) or N (%)
Age at assessment, years	181 358	56.67 (7.91)
Age at menarche, years	176 262	12.95 (1.60)
Age at first sex, years	154 599	19.02 (3.44)
Age first birth, years	124 093	25.39 (4.54)
Age last birth, years	123 926	30.15 (4.80)
Reproductive period, years	123 892	4.76 (3.65)
Number of sexual partners	149 902	4.63 (6.99)
Number of children	181 247	1.81 (1.15)
Childlessness	181 255	
Yes		33 242 (18.34)
No		148 013 (81.66)
Age when left education, years	124 279	16.63 (2.03)
Educational attainment, years	179 731	13.05 (4.32)
Alcohol intake	181 233	
Daily or almost daily		30 918 (17.06)
Three or four times a week		39 346 (21.71)
Once or twice a week		47 864 (26.41)
One to three times a month		23 723 (13.09)
Special occasions only		25 101 (13.85)
Never		14 281 (7.88)
Ever smoked	180 751	
Yes		101 112 (55.94)
No		79 639 (44.06)
Risk taking	174 718	
Yes		31 973 (18.30)
No		142 745 (81.70)

### Age at menarche

Using the 116 SNP instrument for age at menarche we find consistent evidence of a causal effect of earlier age at menarche on earlier age at first birth across all MR methods. We find some evidence of an effect of earlier age at menarche on earlier age at last birth and all MR methods showed point estimates in a consistent direction. There was no clear evidence of an effect of age at menarche on duration of reproductive years, number of children, or number of sexual partners, and little evidence for an effect on likelihood of being childless with results showing confidence intervals consistent with the null and inconsistency for the direction of point estimates across MR approaches. These results are presented in Tables 2 and 3.

**Table 2:** Estimates of the causal effect of earlier age at menarche (116 SNP instrument) on life history outcomes using full UK Biobank data.

		IVW		MR-Egger regression		Weighted median		MBE	
	N	β or OR (95% CI)	*p*	β or OR (95% CI)	*P*	β or OR (95% CI)	*p*	β or OR (95% CI)	*p*
**Reproduction**									
Age first birth	115070–124093	-0.256 (-0.342, -0.171)	<0.001	-0.260 (-0.522, 0.002)	0.05	-0.325 (-0.471, -0.178)	<0.001	-0.362 (-0.722, -0.002)	0.05
Age last birth	114916–123926	-0.235 (-0.325, -0.144)	<0.001	-0.208 (-0.487, 0.07)	0.14	-0.241 (-0.391, -0.091)	0.002	-0.225 (-0.515, 0.066)	0.13
Reproductive period	114883–123892	0.018 (-0.053, 0.088)	0.62	0.040 (-0.175, 0.255)	0.71	0.015 (-0.09, 0.12)	0.78	-0.076 (-0.335, 0.184)	0.57
Number of sexual partners	138920–149902	-0.052 (-0.171, 0.067)	0.39	0.094 (-0.27, 0.459)	0.61	-0.030 (-0.235, 0.175)	0.77	-0.010 (-0.423, 0.403)	0.96
Number of children	168050–181247	-0.016 (-0.034, 0.002)	0.09	0.023 (-0.033, 0.078)	0.42	-0.022 (-0.052, 0.007)	0.14	0.020 (-0.042, 0.082)	0.53
Childlessness	168058–181255	1.060 (1.017, 1.105)	0.01	0.987 (0.869, 1.121)	0.84	1.047 (0.978, 1.121)	0.18	1.038 (0.905, 1.192)	0.59
**Education**									
Age when left education	115204–124279	-0.062 (-0.100, -0.024)	0.002	-0.153 (-0.27, -0.036)	0.01	-0.095 (-0.158, -0.031)	0.004	-0.126 (-0.283, 0.031)	0.12
Educational attainment in years	166640–179731	-0.072 (-0.139, -0.005)	0.04	-0.237 (-0.443, -0.03)	0.03	-0.128 (-0.253, -0.003)	0.05	-0.246 (-0.487, -0.005)	0.05
**Risky behaviours**									
Alcohol intake	168039–181233	0.059 (0.035, 0.083)	<0.001	-0.030 (-0.103, 0.044)	0.43	0.035 (-0.007, 0.077)	0.10	0.007 (-0.075, 0.089)	0.86
Ever smoked	167584–180751	1.002 (0.970, 1.034)	0.92	1.015 (0.921, 1.121)	0.76	0.994 (0.942, 1.049)	0.83	1.035 (0.924, 1.16)	0.55
Risk taking	161994–174718	0.989 (0.949, 1.032)	0.61	1.092 (0.96, 1.242)	0.18	0.984 (0.916, 1.058)	0.67	0.979 (0.832, 1.153)	0.81

Mendelian Randomization approaches used: inverse variance weighted, weighted mode-based estimator, MR-Egger regression and weighted median. (LCI: lower 95% confidence interval; UCI: upper 95% confidence interval; MBE: weighted mode-based estimator).

**Table 3:** MR-Egger intercept values for age at menarche (116 SNP instrument) on life history outcomes using full UK Biobank data.

	MR-Egger intercept		
	β or OR	95% CI	*p*
**Reproduction**			
Age first birth	0.0002	-0.011, 0.012	0.98
Age last birth	-0.001	-0.013, 0.011	0.84
Reproductive period	-0.001	-0.011, 0.008	0.82
Number of sexual partners	-0.007	-0.023, 0.009	0.40
Number of children	-0.002	-0.004, 0.001	0.15
Childlessness	1.003	0.998, 1.009	0.24
**Education**			
Age when left education	0.004	-0.001, 0.009	0.11
Educational attainment in years	0.008	-0.001, 0.017	0.10
**Risky behaviours**			
Alcohol intake	0.004	0.001, 0.007	0.01
Ever smoked	0.999	0.995, 1.004	0.77
Risk taking	0.995	0.990, 1.001	0.11

LCI: lower 95% confidence interval; UCI: upper 95% confidence interval.

For educational outcomes, there was evidence of an effect of earlier age at menarche on lower educational attainment and age at leaving education across most MR methods and consistent point estimates for all MR approaches (Tables 2 and 3). Alcohol intake appeared to decrease with earlier age at menarche, but the MR-Egger intercept (*p* = 0.013) indicated horizontal pleiotropy, suggesting that this effect does not remain when horizontal pleiotropy is accounted for (Tables 2 and 3). No clear evidence was found for effects of age at menarche on having ever smoked or risk taking behaviour although these measures were binary and therefore we had less statistical power to detect effects (Table 2).

After removing SNPs also associated with body mass index [36,44] from our instrument, results were broadly similar to the main analysis although MR-Egger regression analysis showed decreased estimates and for many outcomes the *p*-values increased. This could be due to eliminating a possible pathway via body mass index and/or reduced statistical power as a result of using fewer SNPs (S5 and S6 Tables). We repeated analyses using the 305 SNP instrument for age at menarche. Results were broadly similar to the main analysis (S7–S10 Tables). There was slight increased evidence for an effect on number of sexual partners, ever smoked and childlessness. This analysis suffers from greater bias as it is uses a sub-sample of UK Biobank (described above) or alternatively, when using the entire UK Biobank sample in analyses, it results in overlap between the exposure and outcome datasets, which has shown to bias results towards the observational estimate [35].

### Age at first sexual intercourse

We conducted a fixed effects meta-analysis of the 23 SNP-outcome associations in UK Biobank and found evidence of relationships for earlier age at first sexual intercourse with earlier age at first birth, earlier age at last birth, a longer reproductive period, increased number of sexual partners, a greater number of children, decreased likelihood of being childlessness, earlier age at leaving education, lower educational attainment, increased likelihood of having ever smoked and increased likelihood of risk taking behaviour (S11 Table).

We also used MR to examine effects between age at first sexual intercourse and these life history outcomes, taking SNP-exposure associations from a GWAS [27] and SNP-outcome associations in a sub-sample of UK Biobank, therefore likely affected by selection bias. There appeared to be a consistent effect of earlier age at first sexual intercourse on earlier age at last birth and increased likelihood of risk taking behaviour across MR methods (S12 Table). However, the MR-Egger intercept showed evidence of horizontal pleiotropy for most outcomes and, as discussed above, this MR analysis may suffer from bias due to stratifying the UK Biobank sample (S13 Table). Results for Radial MR and a leave one out analysis suggested no strong influence of outliers (further details are provided in S1 Text and S1 Fig).

## Discussion

We find evidence for causal relationships between earlier age at menarche and earlier age at first birth, earlier age at last birth, lower educational attainment, and earlier age at leaving education. Results for effects of earlier age at menarche on lower educational outcomes are consistent with previous findings [27,45]. We found no clear effect of age at menarche on number of children in this female only sample, supporting previous findings for females [27]. Here, applying additional MR methods to those used previously, we find that the effect of age at menarche on alcohol intake is not robust [27]. Results for educational attainment, age at first birth and age at last birth were as expected and suggest that earlier age at menarche is causally related to some traits characterizing a fast life history strategy. Possible reasons for lack of evidence between age at menarche and other outcomes, such as reproductive period, are discussed below.

Evidence for age at first sexual intercourse on these life history traits was mixed across analyses, with MR results suggesting possible violation of the exclusion restriction assumption of no direct effects of the instrument on the outcome not acting through the exposure (i.e., the presence of horizontal pleiotropy) [15,23]. We detect the presence of horizontal pleiotropy on multiple outcomes and we therefore cannot infer causality from this analysis. Interestingly, these results suggest that previous findings may have also included pleiotropic effects and may be questionable [27]. Results for age at first sexual intercourse are therefore not robust. These results provide a valuable proof of concept for using MR to test evolutionary theory, but cannot be used to infer causal effects of age at first sexual intercourse. When further GWAS on age at first sexual intercourse become available, it will be possible to repeat investigation of horizontal pleiotropic effects and overcome other limitations of this analysis discussed below.

The effects of earlier age at menarche on these reproductive and educational traits can be viewed as directing effort towards short-term reproductive goals and risky behaviour as an important part of a fast life history strategy [1]. Variation in age at menarche may therefore represent an important causal component of a suite of adaptations [6]. Earlier age at first birth can be considered an adaptive response to early life adversity and our finding of an effect of earlier age at menarche on earlier age at first birth is therefore in line with this [46]. It is, however, interesting that we see an effect of earlier age at menarche on earlier age at last birth, with no clear effect on reproductive period. This suggests that individuals on a fast life history strategy are not just starting their reproductive life earlier but shifting their reproductive life forward in time. Nettle highlights that individuals in more deprived areas with short life expectancy, likely on a fast life history strategy, need to reproduce earlier than individuals in more affluent areas with higher life expectancy to be in good health for an equivalent period of care [47]. This finding of a causal relationship between age at menarche and age at first and last birth is also important for research investigating the effects of reproductive timings on later life health outcomes [48]. Education is a key predictor of positive later life outcomes in the UK, and our finding of a causal effect of earlier age at menarche on decreased educational attainment provides important information for determinants of educational attainment which should be independent from confounding [45]. Investing in education can be seen as a slow life history trait with delayed benefits [49]. The effect of age at menarche on educational attainment may be due to variation in cognition following variation in age at menarche and gonadal hormones, due to menarche, that may influence behaviour during schooling [45,50]. Although we cannot easily intervene on age at menarche, if factors on the causal pathway from age at menarche to outcomes are modifiable (e.g., behaviour during schooling) these could provide targets for interventions.

As a component of life history strategy, we would have expected to see an effect of earlier age at menarche on increased number of children or likelihood of remaining childless, although access to contraception may influence this relationship. Number of sexual partners has previously been used as a proxy for reproductive success in a post-contraceptive environment, although it should be noted that contraception allows for the decoupling of sexual and reproductive partners [51]. We did not find a clear effect of age at menarche on number of sexual partners although female reproductive success is less dependent on number of sexual partners than males. It is further possible that the effect of menarche on number of children is masked by the detrimental effects of risky behaviours, such as substance use, on fertility in the modern environment [52,53]. Although our results show no clear evidence of an effect of earlier age at menarche on increased risky behaviours and substance use, binary measures of smoking and risk taking were used, resulting in less statistical power. Furthermore, the measure of risk taking was a single item asking whether participants would describe themselves as someone who takes risks and may not capture the full extent of risk taking behaviour. We did not show an effect of age at menarche on alcohol intake, another form of substance use which has also been shown to be associated with decreased fertility [52,53]. Further research should examine the mediating causal relationships between age at menarche and fertility in the modern environment using more detailed measures of substance use and larger samples.

Our study highlights how MR can be applied to test predictions within life history theory to provide evidence of causality and increase our understanding of health and social behaviour. A strength of the present study is the use of multiple MR methods. This allowed us to extend upon the findings of previous research [27] and triangulate across methods, each with varying and orthogonal assumptions, to provide greater confidence in results [54]. We were further able to compare evidence using two instruments for age at menarche. Additionally, we used a large population-based sample for our analysis to help identify the small effects common in genetic studies [38], although we acknowledge that for binary outcomes power was more limited.

There are currently no strong instruments for early life adversity and it is therefore not easily possible to test the causal effects of early life adversity on age at menarche and age at first sexual intercourse using MR [55]. This aspect of life history theory has been the focus of most prior work. However, we examined the effects of two intermediate reproductive traits (age at menarche and age at first sexual intercourse) on further reproductive and behavioural outcomes. Early menarche is associated with both good condition and early life adversity, likely with different developmental pathways. We did not stratify analysis on any measure of adversity, or a proxy for adversity such as socioeconomic status. There has been a secular trend of decreasing age at menarche in recent times, perhaps due to increasing levels of obesity or improved living conditions [2]. This trend therefore also includes individuals that are assumed to be on a slow life history strategy. The present study therefore cannot fully disentangle those on a fast or slow life history strategy although it is assumed that earlier menarche and age at first sexual intercourse can be used as an indicator of an individual being on a fast life history strategy. We attempted to account for the possibility of effects of age at menarche acting via BMI in sensitivity analyses. The multiple possible interpretations of early age at menarche and age at first sexual intercourse are a strong limitation of this work however it is still important to examine all components of the life history theory framework, rather than to focus only on the effects of early life adversity on reproductive traits such as age at menarche.

Other limitations of our work should be considered. First, the age at first sexual intercourse GWAS was conducted in a sub-sample of UK Biobank data and we therefore conducted an unweighted analysis due to this sample overlap, using a fixed effects meta-analysis method. We additionally conducted MR by dividing our outcome sample to avoid overlap of participants, however this may have introduced bias as the sub-division is related to smoking status and therefore akin to stratifying on smoking, which may be affected by our exposure and outcome (termed collider bias) [34]. Second, given the MR results between age at menarche and educational attainment, it is possible that parents could pass on genotypes for age at menarche and educational attainment and therefore create confounding by parental genotype. This type of genetic confounding is possible in MR studies and unfortunately cannot be tested or addressed with UK Biobank data due to having genotype data on only one generation. Third, we used SNPs for age at first sexual intercourse, and their associations, identified in pooled sex GWAS, due to reductions in power of using SNPs identified in females only and our exposure and outcome data therefore consists of different populations (not advised for MR studies) [24]. Although most variants showed sex-concordant associations in the GWAS, six variants in our instrument for age at first sexual intercourse showed some evidence of sex-discordant associations [27]. Fourth, the SNP-exposure associations were used from discovery analysis, which may cause upward bias of estimates [36,56], however using discovery data is common in MR studies and our unweighted analysis for age at first sexual intercourse did not use GWAS estimates [36,57]. Fifth, UK Biobank data is unrepresentative of the population, with a 5% response rate, and therefore and suffers from selection bias which may generate spurious associations [30,58,59]. Finally, variants are non-specific and we cannot fully remove population structure, which can induce spurious associations through confounding, even within a sample of European ancestry and adjusting for principal components as done so here [60].

## Conclusions

We found some evidence that age at menarche is causally related to other life history traits and outcomes. Age at first sexual intercourse was also related to many life history outcomes, although there was evidence of horizontal pleiotropy which violates the exclusion restriction assumption of MR and these results are therefore an interesting proof of concept for using MR to test evolutionary theory and in light of previous results, cannot be used to infer causal effects of age at first sexual intercourse. [22,23]. The age at menarche results highlight how analysis techniques from genetic epidemiology can be used to answer how life history traits are related within life history strategies, and to better understand determinants of health and social behaviour. There are increasing numbers of GWAS conducted on evolutionary relevant traits and we have demonstrated that future research could apply these MR techniques to further test predictions of life history theory.

## Supporting information

S1 Text (DOCX)

S1 Data (TMP)

S1 Fig Leave-one-out analysis indicates that all estimates were within the confidence intervals of all other estimates. Here shown for increasing age at first sexual intercourse on age at first birth.
(DOCX)

S1 Table List of SNPs used in analysis and their associations with age at menarche (*p*<5×10^−8^) from Perry et al. (1). (DOCX)

S2 Table List of SNPs used in analysis and their associations with age at menarche (*p*<5×10^−8^) from Day et al. (2). (DOCX)

S3 Table List of SNPs used in analysis and their associations with age at first sexual intercourse (*p*< 5×10^−8^) from Day et al. (3). (DOCX)

S4 Table Estimates for the mean F statistic, I2GX, and Cochran’s Q. (DOCX)

S5 Table Estimates of the causal effect of earlier age at menarche (116 SNPs) on life history outcomes using full UK Biobank data excluding SNPs associated with body mass index at *p*<5×10^−8^ (9 SNPs excluded). (DOCX)

S6 Table MR-Egger intercept values for age at menarche (116 SNPs) on life history outcomes using full UK Biobank data and excluding SNPs associated with body mass index at *p*<5×10^−8^ (9 SNPs excluded). (DOCX)

S7 Table Estimates of the causal effect of earlier age at menarche (305 SNPs) on life history outcomes using non-overlapping UK Biobank data. (DOCX)

S8 Table MR-Egger intercept values for age at menarche (305 SNPs) on life history outcomes using non-overlapping UK Biobank data. (DOCX)

S9 Table Estimates of the causal effect of earlier age at menarche (305 SNPs) on life history outcomes using full UK Biobank data. (DOCX)

S10 Table MR-Egger intercept values for age at menarche (305 SNPs) on life history outcomes using full UK Biobank data. (DOCX)

S11 Table Fixed effects meta-analysis of SNP-outcome associations using full UK Biobank and SNPs identified for age at first sexual intercourse (23 SNPs). (DOCX)

S12 Table Estimates of the causal effect of earlier age at first sexual intercourse on life history outcomes using non-overlapping UK Biobank data. (DOCX)

S13 Table SIMEX unweighted MR-Egger intercept values for age at first sexual intercourse on life history outcomes using non-overlapping UK Biobank data. (DOCX)
