# Supplementary material for: The effects of age at menarche and first sexual intercourse on reproductive and behavioural outcomes: A Mendelian randomization study
Source: PLoS One. 2020 Jun 15;15(6):e0234488. doi: 10.1371/journal.pone.0234488 (PMC7295202; doi:10.1371/journal.pone.0234488)
Supplement: S1 Text — (DOCX) [file pone.0234488.s001.docx]

**Supplementary Text**

**Methods**

For our age at menarche instrument, where palindromic SNPs were used, the minor allele frequency was checked to ensure there were no issues with strand mismatches and no palindromic SNPs with minor allele frequency around 0.5 were used, where the strand is difficult to identify. One of the 123 SNPs was removed due to high instability in its estimates. This resulted in 116 and 305 SNPs as instruments for age at menarche that were available in our outcome sample (UK Biobank), excluding compound and tricyclic SNPs. Of the 33 SNPs for our second instrument of age at first sexual intercourse, there were 23 SNPs available in our outcome sample (UK Biobank), with no palindromic SNPs having minor allele frequencies around 0.5.

**Results**

There appeared to be a consistent outlier in age at first sexual intercourse analysis (rs538498277) when plotting the but there was no formal evidence of this using radial MR, with second order modified weights and a p-value of 0.01, apart from in relation to ever smoked as an outcome. Another SNP (rs2188151) was most often identified as the top outlier. We therefore conducted a leave one out analysis to ensure that no outliers were having a relatively large effect on estimates which showed that estimates with a SNP removed were all within the confidence intervals for every other SNP, suggesting no strong influence of outliers (See Supplementary Figure 1 for example with age at first birth).
