# Supplementary material for: The effects of age at menarche and first sexual intercourse on reproductive and behavioural outcomes: A Mendelian randomization study
Source: PLoS One. 2020 Jun 15;15(6):e0234488. doi: 10.1371/journal.pone.0234488 (PMC7295202; doi:10.1371/journal.pone.0234488)

**Figure S1.** Leave-one-out analysis indicates that all estimates were within the confidence intervals of all other estimates. Here shown for increasing age at first sexual intercourse on age at first birth.


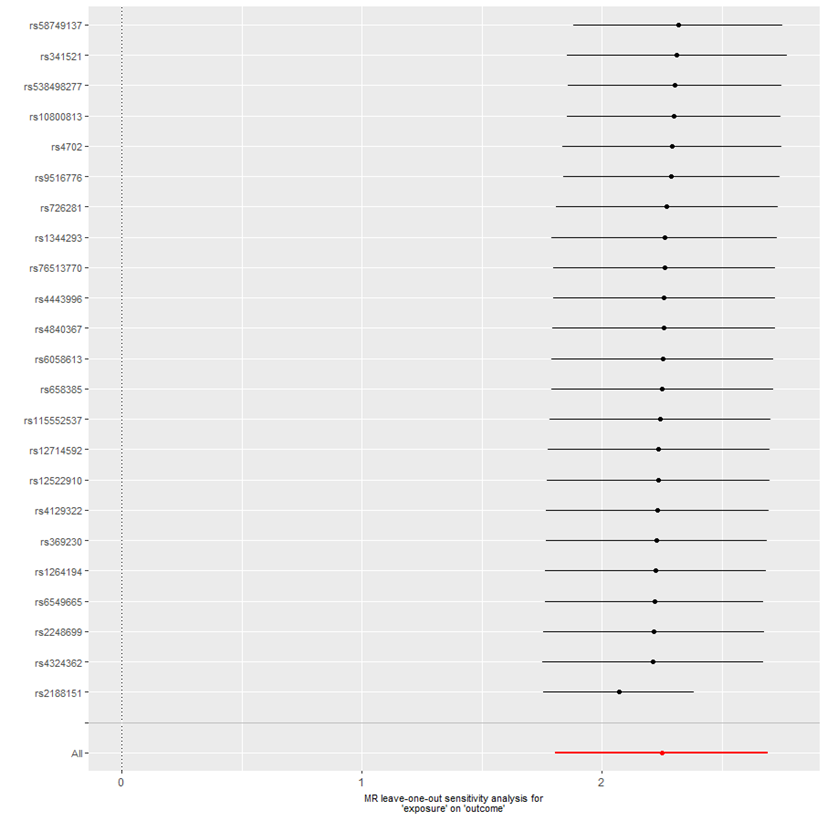

Supplement: S1 Fig — Here shown for increasing age at first sexual intercourse on age at first birth. (DOCX) [file pone.0234488.s003.docx]
