# Supplementary material for: The effects of age at menarche and first sexual intercourse on reproductive and behavioural outcomes: A Mendelian randomization study
Source: PLoS One. 2020 Jun 15;15(6):e0234488. doi: 10.1371/journal.pone.0234488 (PMC7295202; doi:10.1371/journal.pone.0234488)
Supplement: S1 Table — (DOCX) [file pone.0234488.s004.docx]

**Table S1.** List of SNPs used in analysis and their associations with age at menarche (*p*<5×10^-8^) from Perry et al. (1)

| **SNP** | **β** | **SE** |
| --- | --- | --- |
| rs10144321 | 0.04 | 0.006 |
| rs1038903 | 0.04 | 0.006 |
| rs10423674 | 0.04 | 0.005 |
| rs10453225 | 0.09 | 0.005 |
| rs10739221 | 0.08 | 0.006 |
| rs10789181 | 0.03 | 0.005 |
| rs1079866 | 0.07 | 0.007 |
| rs10816359 | 0.04 | 0.008 |
| rs10895140 | 0.04 | 0.005 |
| rs10938397 | 0.04 | 0.005 |
| rs10980854 | 0.06 | 0.011 |
| rs10980921 | 0.09 | 0.009 |
| rs11022756 | 0.05 | 0.006 |
| rs11165924 | 0.03 | 0.006 |
| rs11215400 | 0.04 | 0.006 |
| rs1129700 | 0.03 | 0.005 |
| rs11578152 | 0.03 | 0.005 |
| rs11715566 | 0.05 | 0.005 |
| rs11767400 | 0.04 | 0.006 |
| rs11792861 | 0.04 | 0.005 |
| rs12148769 | 0.05 | 0.008 |
| rs12446632 | 0.04 | 0.007 |
| rs12472911 | 0.04 | 0.006 |
| rs1254337 | 0.04 | 0.005 |
| rs12571664 | 0.04 | 0.006 |
| rs12607903 | 0.04 | 0.005 |
| rs12915845 | 0.03 | 0.005 |
| rs13053505 | 0.04 | 0.007 |
| rs13067731 | 0.04 | 0.007 |
| rs13179411 | 0.06 | 0.007 |
| rs13196561 | 0.04 | 0.006 |
| rs1324913 | 0.03 | 0.005 |
| rs1364063 | 0.05 | 0.005 |
| rs1400974 | 0.05 | 0.005 |
| rs1461503 | 0.05 | 0.005 |
| rs1469039 | 0.05 | 0.007 |
| rs1532331 | 0.03 | 0.005 |
| rs16860328 | 0.04 | 0.005 |
| rs16896742 | 0.04 | 0.005 |
| rs16918254 | 0.05 | 0.009 |
| rs16918636 | 0.03 | 0.006 |
| rs17086188 | 0.07 | 0.013 |
| rs17171818 | 0.04 | 0.006 |
| rs17233066 | 0.09 | 0.014 |
| rs17236969 | 0.05 | 0.008 |
| rs17266097 | 0.04 | 0.005 |
| rs1915146 | 0.03 | 0.005 |
| rs1958560 | 0.03 | 0.005 |
| rs2063730 | 0.05 | 0.007 |
| rs2137289 | 0.05 | 0.005 |
| rs2153127 | 0.08 | 0.005 |
| rs2274465 | 0.03 | 0.005 |
| rs239198 | 0.03 | 0.005 |
| rs244293 | 0.03 | 0.005 |
| rs246185 | 0.04 | 0.006 |
| rs2479724 | 0.03 | 0.005 |
| rs251130 | 0.04 | 0.006 |
| rs2600959 | 0.04 | 0.005 |
| rs268067 | 0.04 | 0.006 |
| rs2687729 | 0.04 | 0.006 |
| rs2688325 | 0.03 | 0.006 |
| rs2947411 | 0.06 | 0.007 |
| rs3101336 | 0.04 | 0.005 |
| rs3733631 | 0.05 | 0.007 |
| rs3743266 | 0.04 | 0.005 |
| rs4369815 | 0.06 | 0.01 |
| rs466639 | 0.08 | 0.007 |
| rs4840086 | 0.04 | 0.005 |
| rs4895808 | 0.03 | 0.005 |
| rs543874 | 0.05 | 0.006 |
| rs6009583 | 0.03 | 0.006 |
| rs6427782 | 0.03 | 0.005 |
| rs652260 | 0.03 | 0.005 |
| rs6555855 | 0.04 | 0.006 |
| rs6563739 | 0.03 | 0.005 |
| rs6747380 | 0.07 | 0.007 |
| rs6758290 | 0.04 | 0.005 |
| rs6762477 | 0.04 | 0.006 |
| rs6770162 | 0.04 | 0.005 |
| rs6933660 | 0.03 | 0.005 |
| rs6938574 | 0.04 | 0.007 |
| rs6964833 | 0.04 | 0.006 |
| rs7037266 | 0.03 | 0.005 |
| rs7103411 | 0.04 | 0.006 |
| rs7104764 | 0.03 | 0.006 |
| rs7138803 | 0.04 | 0.005 |
| rs7141210 | 0.03 | 0.005 |
| rs7215990 | 0.04 | 0.006 |
| rs7463166 | 0.03 | 0.005 |
| rs7514705 | 0.04 | 0.005 |
| rs7642134 | 0.04 | 0.005 |
| rs7647973 | 0.05 | 0.006 |
| rs7701886 | 0.03 | 0.005 |
| rs7759938 | 0.12 | 0.005 |
| rs7821178 | 0.04 | 0.005 |
| rs7828501 | 0.04 | 0.005 |
| rs7853970 | 0.03 | 0.005 |
| rs7865468 | 0.03 | 0.005 |
| rs7955374 | 0.04 | 0.008 |
| rs8032675 | 0.04 | 0.005 |
| rs8050136 | 0.04 | 0.005 |
| rs852069 | 0.04 | 0.005 |
| rs889122 | 0.04 | 0.006 |
| rs900400 | 0.03 | 0.005 |
| rs913588 | 0.03 | 0.005 |
| rs929843 | 0.04 | 0.006 |
| rs9321659 | 0.06 | 0.008 |
| rs939317 | 0.04 | 0.006 |
| rs9447700 | 0.03 | 0.005 |
| rs9475752 | 0.04 | 0.006 |
| rs951366 | 0.03 | 0.005 |
| rs9560113 | 0.05 | 0.006 |
| rs9635759 | 0.05 | 0.005 |
| rs9647570 | 0.05 | 0.007 |
| rs9849248 | 0.04 | 0.007 |
| rs988913 | 0.04 | 0.005 |
