# Supplementary material for: The effects of age at menarche and first sexual intercourse on reproductive and behavioural outcomes: A Mendelian randomization study
Source: PLoS One. 2020 Jun 15;15(6):e0234488. doi: 10.1371/journal.pone.0234488 (PMC7295202; doi:10.1371/journal.pone.0234488)
Supplement: S2 Table — (DOCX) [file pone.0234488.s005.docx]

**Table S2.** List of SNPs used in analysis and their associations with age at menarche (*p*<5×10^-8^) from Day et al. (2)

| **SNP** | **β** | **SE** |
| --- | --- | --- |
| rs10136330 | -0.06 | 0.010 |
| rs10138913 | 0.06 | 0.004 |
| rs10143972 | -0.04 | 0.005 |
| rs10145469 | -0.06 | 0.009 |
| rs10156597 | 0.10 | 0.004 |
| rs10175423 | -0.02 | 0.004 |
| rs10205969 | -0.04 | 0.005 |
| rs10237306 | 0.03 | 0.004 |
| rs1023955 | -0.03 | 0.004 |
| rs10268051 | 0.02 | 0.005 |
| rs1030015 | -0.02 | 0.004 |
| rs1032682 | 0.02 | 0.004 |
| rs10400136 | -0.03 | 0.004 |
| rs10422323 | 0.04 | 0.006 |
| rs10521021 | -0.02 | 0.004 |
| rs1054442 | 0.04 | 0.004 |
| rs10750766 | -0.03 | 0.004 |
| rs10782777 | -0.03 | 0.004 |
| rs1079866 | -0.07 | 0.006 |
| rs10832021 | -0.05 | 0.004 |
| rs10885077 | 0.02 | 0.004 |
| rs10906395 | -0.02 | 0.004 |
| rs10931831 | -0.05 | 0.004 |
| rs10933 | -0.02 | 0.004 |
| rs10934420 | -0.05 | 0.004 |
| rs10959016 | -0.03 | 0.005 |
| rs10959552 | -0.04 | 0.006 |
| rs10978641 | -0.03 | 0.005 |
| rs10992769 | 0.03 | 0.004 |
| rs11031040 | -0.04 | 0.005 |
| rs11065822 | 0.03 | 0.004 |
| rs11079810 | 0.04 | 0.006 |
| rs11165924 | 0.03 | 0.004 |
| rs11209331 | 0.02 | 0.004 |
| rs11209943 | 0.04 | 0.004 |
| rs11210871 | 0.04 | 0.004 |
| rs11240695 | -0.03 | 0.004 |
| rs112991346 | -0.04 | 0.006 |
| rs113388806 | -0.06 | 0.010 |
| rs1148006 | -0.03 | 0.004 |
| rs115260227 | -0.16 | 0.024 |
| rs11534296 | -0.04 | 0.004 |
| rs115435316 | 0.11 | 0.011 |
| rs11556924 | 0.02 | 0.004 |
| rs11606190 | 0.04 | 0.006 |
| rs11619721 | -0.04 | 0.007 |
| rs11668587 | -0.03 | 0.004 |
| rs11711674 | 0.02 | 0.004 |
| rs117143374 | -0.05 | 0.006 |
| rs1172955 | -0.04 | 0.004 |
| rs117530880 | -0.07 | 0.012 |
| rs11756746 | 0.02 | 0.005 |
| rs11767400 | 0.03 | 0.004 |
| rs11786868 | 0.03 | 0.005 |
| rs11792861 | 0.03 | 0.004 |
| rs11852771 | 0.02 | 0.004 |
| rs11873906 | -0.05 | 0.004 |
| rs12040029 | -0.04 | 0.006 |
| rs12125335 | -0.05 | 0.006 |
| rs12200565 | 0.03 | 0.004 |
| rs12460047 | -0.03 | 0.004 |
| rs12467441 | -0.04 | 0.006 |
| rs12571664 | 0.04 | 0.005 |
| rs12603280 | -0.04 | 0.005 |
| rs12663002 | 0.04 | 0.006 |
| rs12894936 | -0.05 | 0.004 |
| rs12915845 | -0.04 | 0.004 |
| rs12937034 | -0.03 | 0.004 |
| rs13023912 | -0.05 | 0.004 |
| rs13043968 | -0.04 | 0.006 |
| rs13120031 | 0.03 | 0.004 |
| rs13199764 | 0.04 | 0.005 |
| rs13233916 | -0.05 | 0.008 |
| rs13278754 | -0.03 | 0.004 |
| rs13283567 | -0.04 | 0.006 |
| rs1329767 | -0.03 | 0.004 |
| rs13322435 | 0.04 | 0.004 |
| rs1414186 | -0.04 | 0.005 |
| rs141847393 | 0.04 | 0.007 |
| rs142058842 | -0.07 | 0.005 |
| rs142643995 | 0.06 | 0.012 |
| rs1428120 | 0.03 | 0.004 |
| rs1435753 | -0.03 | 0.004 |
| rs1449543 | 0.02 | 0.004 |
| rs145438026 | -0.07 | 0.008 |
| rs1456031 | 0.02 | 0.004 |
| rs150821390 | 0.07 | 0.012 |
| rs1512238 | -0.05 | 0.004 |
| rs151680 | 0.03 | 0.004 |
| rs1535252 | -0.03 | 0.004 |
| rs153793 | -0.02 | 0.004 |
| rs1539310 | 0.02 | 0.005 |
| rs1566385 | 0.06 | 0.008 |
| rs15671 | -0.02 | 0.004 |
| rs1571536 | 0.03 | 0.004 |
| rs157877 | -0.08 | 0.006 |
| rs1601615 | -0.03 | 0.004 |
| rs16841867 | 0.05 | 0.006 |
| rs169080 | -0.03 | 0.004 |
| rs16917237 | 0.04 | 0.005 |
| rs16918378 | 0.05 | 0.006 |
| rs16937956 | -0.04 | 0.004 |
| rs17035311 | 0.04 | 0.005 |
| rs1704528 | -0.05 | 0.004 |
| rs17171852 | -0.04 | 0.005 |
| rs17390720 | 0.03 | 0.004 |
| rs17563472 | -0.06 | 0.011 |
| rs17564430 | -0.04 | 0.004 |
| rs1815811 | -0.03 | 0.004 |
| rs184033703 | -0.05 | 0.009 |
| rs1885740 | -0.03 | 0.005 |
| rs1925047 | -0.03 | 0.004 |
| rs1971554 | 0.03 | 0.004 |
| rs1984870 | 0.04 | 0.004 |
| rs2066323 | -0.02 | 0.004 |
| rs2108753 | 0.03 | 0.004 |
| rs222440 | -0.03 | 0.005 |
| rs2267812 | 0.04 | 0.005 |
| rs2271758 | -0.02 | 0.004 |
| rs2295094 | 0.04 | 0.005 |
| rs2300922 | 0.04 | 0.004 |
| rs2312205 | 0.03 | 0.005 |
| rs2343507 | 0.02 | 0.004 |
| rs2378100 | -0.02 | 0.004 |
| rs2461794 | 0.03 | 0.004 |
| rs247520 | 0.04 | 0.005 |
| rs2546959 | 0.03 | 0.005 |
| rs2558101 | -0.02 | 0.004 |
| rs256350 | -0.02 | 0.004 |
| rs2604265 | 0.04 | 0.004 |
| rs2659007 | -0.03 | 0.004 |
| rs2661339 | 0.05 | 0.009 |
| rs2679894 | 0.05 | 0.004 |
| rs2688326 | -0.04 | 0.004 |
| rs2723065 | -0.02 | 0.004 |
| rs2724961 | -0.05 | 0.004 |
| rs2770957 | 0.03 | 0.005 |
| rs2780243 | -0.02 | 0.004 |
| rs28757192 | -0.06 | 0.011 |
| rs2889128 | 0.02 | 0.004 |
| rs29941 | 0.03 | 0.004 |
| rs3021057 | 0.02 | 0.004 |
| rs3113862 | -0.04 | 0.004 |
| rs34437050 | 0.24 | 0.020 |
| rs34513772 | 0.02 | 0.004 |
| rs35436838 | -0.07 | 0.011 |
| rs35485457 | -0.04 | 0.004 |
| rs35935052 | 0.04 | 0.005 |
| rs360495 | 0.04 | 0.007 |
| rs36093651 | 0.04 | 0.005 |
| rs3733632 | -0.05 | 0.005 |
| rs3743266 | 0.04 | 0.004 |
| rs3746037 | 0.04 | 0.005 |
| rs3746619 | 0.05 | 0.007 |
| rs3764002 | -0.03 | 0.005 |
| rs3782120 | 0.03 | 0.004 |
| rs3809624 | -0.03 | 0.004 |
| rs3815212 | 0.03 | 0.005 |
| rs395962 | 0.13 | 0.004 |
| rs4303811 | -0.04 | 0.006 |
| rs4327718 | -0.03 | 0.005 |
| rs4340786 | 0.04 | 0.004 |
| rs4359170 | 0.03 | 0.004 |
| rs437836 | 0.04 | 0.005 |
| rs443252 | 0.06 | 0.009 |
| rs4448948 | -0.04 | 0.008 |
| rs446745 | -0.03 | 0.005 |
| rs4487799 | 0.02 | 0.004 |
| rs4561063 | 0.03 | 0.004 |
| rs4588499 | -0.02 | 0.004 |
| rs467379 | 0.02 | 0.004 |
| rs4701140 | 0.02 | 0.004 |
| rs474463 | -0.03 | 0.005 |
| rs4746113 | -0.02 | 0.004 |
| rs4751614 | 0.03 | 0.005 |
| rs4778356 | 0.04 | 0.006 |
| rs4801809 | -0.04 | 0.007 |
| rs4804025 | -0.04 | 0.004 |
| rs4813429 | 0.03 | 0.005 |
| rs4836984 | 0.03 | 0.004 |
| rs484353 | 0.03 | 0.004 |
| rs4845364 | 0.02 | 0.004 |
| rs4859001 | 0.04 | 0.006 |
| rs4875424 | -0.03 | 0.004 |
| rs4877387 | 0.02 | 0.004 |
| rs4886140 | 0.03 | 0.004 |
| rs4945266 | -0.04 | 0.005 |
| rs4951261 | 0.03 | 0.004 |
| rs4970598 | 0.06 | 0.011 |
| rs4976623 | 0.03 | 0.005 |
| rs506589 | 0.07 | 0.005 |
| rs552491 | -0.03 | 0.004 |
| rs55680968 | -0.05 | 0.008 |
| rs55784701 | 0.03 | 0.005 |
| rs56367141 | -0.04 | 0.006 |
| rs56409371 | -0.03 | 0.005 |
| rs5742915 | -0.02 | 0.004 |
| rs5753377 | -0.03 | 0.004 |
| rs582780 | 0.03 | 0.004 |
| rs59246405 | 0.03 | 0.004 |
| rs59543819 | -0.03 | 0.004 |
| rs59652033 | -0.03 | 0.004 |
| rs61817552 | -0.03 | 0.005 |
| rs61828391 | -0.03 | 0.006 |
| rs61846901 | -0.03 | 0.004 |
| rs6185 | -0.03 | 0.004 |
| rs62104180 | 0.11 | 0.010 |
| rs62229372 | 0.05 | 0.006 |
| rs62316795 | 0.04 | 0.005 |
| rs62342064 | 0.06 | 0.007 |
| rs62361685 | 0.05 | 0.009 |
| rs62379978 | -0.06 | 0.005 |
| rs62391851 | -0.06 | 0.009 |
| rs6415872 | 0.02 | 0.004 |
| rs6434162 | -0.04 | 0.005 |
| rs643428 | -0.02 | 0.004 |
| rs6439371 | -0.03 | 0.004 |
| rs6439713 | 0.03 | 0.004 |
| rs6445624 | 0.04 | 0.006 |
| rs6575806 | -0.03 | 0.006 |
| rs6590889 | -0.04 | 0.004 |
| rs660549 | -0.02 | 0.004 |
| rs66508321 | -0.03 | 0.004 |
| rs6661100 | 0.05 | 0.007 |
| rs6678140 | -0.03 | 0.004 |
| rs6735626 | 0.02 | 0.004 |
| rs68002803 | 0.03 | 0.004 |
| rs6803264 | 0.03 | 0.005 |
| rs6864818 | 0.04 | 0.005 |
| rs6878910 | 0.04 | 0.006 |
| rs6911407 | 0.03 | 0.004 |
| rs6911527 | 0.03 | 0.005 |
| rs6927679 | 0.03 | 0.004 |
| rs6931884 | 0.06 | 0.006 |
| rs6933660 | -0.03 | 0.004 |
| rs7072571 | 0.03 | 0.006 |
| rs7077302 | 0.05 | 0.007 |
| rs709488 | -0.02 | 0.004 |
| rs7108556 | 0.03 | 0.005 |
| rs7115444 | 0.03 | 0.005 |
| rs7132908 | -0.04 | 0.004 |
| rs7178532 | 0.04 | 0.004 |
| rs7218751 | 0.03 | 0.005 |
| rs7239114 | -0.02 | 0.004 |
| rs72756954 | 0.06 | 0.008 |
| rs72787511 | 0.06 | 0.011 |
| rs72842141 | -0.06 | 0.009 |
| rs73035994 | -0.09 | 0.012 |
| rs73187215 | -0.04 | 0.007 |
| rs73435048 | -0.04 | 0.008 |
| rs7359336 | -0.05 | 0.004 |
| rs73820560 | -0.03 | 0.006 |
| rs7426534 | -0.02 | 0.004 |
| rs7431217 | 0.02 | 0.004 |
| rs74499585 | 0.06 | 0.008 |
| rs7516763 | 0.02 | 0.004 |
| rs7542538 | 0.03 | 0.005 |
| rs7576624 | -0.07 | 0.005 |
| rs758747 | -0.03 | 0.004 |
| rs7587651 | -0.02 | 0.004 |
| rs7649124 | 0.03 | 0.005 |
| rs7712046 | -0.03 | 0.004 |
| rs77530428 | -0.12 | 0.017 |
| rs77532868 | 0.06 | 0.010 |
| rs7753896 | 0.03 | 0.004 |
| rs7757654 | -0.03 | 0.004 |
| rs77955256 | -0.04 | 0.006 |
| rs7826872 | 0.03 | 0.004 |
| rs7849973 | 0.02 | 0.004 |
| rs7852169 | -0.10 | 0.007 |
| rs7853970 | 0.04 | 0.004 |
| rs78928932 | -0.06 | 0.009 |
| rs7907759 | 0.04 | 0.004 |
| rs79084266 | -0.04 | 0.007 |
| rs7912468 | -0.02 | 0.004 |
| rs79541760 | 0.04 | 0.005 |
| rs7971408 | 0.05 | 0.006 |
| rs7979001 | 0.02 | 0.004 |
| rs80170948 | -0.07 | 0.011 |
| rs8040272 | 0.04 | 0.006 |
| rs8051833 | -0.04 | 0.004 |
| rs813301 | 0.03 | 0.004 |
| rs8136272 | 0.04 | 0.004 |
| rs842567 | -0.03 | 0.005 |
| rs852061 | -0.04 | 0.004 |
| rs910425 | -0.02 | 0.004 |
| rs913588 | -0.03 | 0.004 |
| rs9330454 | -0.03 | 0.004 |
| rs9349203 | -0.04 | 0.004 |
| rs9382676 | 0.04 | 0.005 |
| rs9403051 | 0.04 | 0.004 |
| rs941520 | -0.02 | 0.004 |
| rs9427116 | 0.02 | 0.004 |
| rs953230 | 0.03 | 0.004 |
| rs9548873 | -0.03 | 0.004 |
| rs9568123 | -0.03 | 0.005 |
| rs9614460 | -0.02 | 0.004 |
| rs9635759 | 0.06 | 0.004 |
| rs9647570 | -0.04 | 0.006 |
| rs970179 | 0.02 | 0.004 |
| rs975642 | -0.02 | 0.004 |
| rs9758500 | -0.05 | 0.004 |
| rs9834893 | -0.05 | 0.007 |
| rs9972653 | -0.05 | 0.004 |
| rs999885 | 0.02 | 0.004 |
