# Supplementary material for: The effects of age at menarche and first sexual intercourse on reproductive and behavioural outcomes: A Mendelian randomization study
Source: PLoS One. 2020 Jun 15;15(6):e0234488. doi: 10.1371/journal.pone.0234488 (PMC7295202; doi:10.1371/journal.pone.0234488)
Supplement: S3 Table — (DOCX) [file pone.0234488.s006.docx]

**Table S3.** List of SNPs used in analysis and their associations with age at first sexual intercourse (*p*< 5×10^-8^) from Day et al. (3)

| **SNP** | **β** | **SE** |
| --- | --- | --- |
| rs10800813 | 0.02 | 0.004 |
| rs115552537 | 0.03 | 0.005 |
| rs12522910 | 0.04 | 0.005 |
| rs1264194 | 0.03 | 0.004 |
| rs12714592 | 0.03 | 0.004 |
| rs1344293 | 0.03 | 0.004 |
| rs2188151 | 0.03 | 0.004 |
| rs2248699 | 0.02 | 0.004 |
| rs341521 | 0.03 | 0.004 |
| rs369230 | 0.03 | 0.004 |
| rs4129322 | 0.04 | 0.007 |
| rs4324362 | 0.03 | 0.004 |
| rs4443996 | 0.02 | 0.004 |
| rs4702 | 0.02 | 0.004 |
| rs4840367 | 0.03 | 0.004 |
| rs538498277 | 0.31 | 0.051 |
| rs58749137 | 0.02 | 0.004 |
| rs6058613 | 0.03 | 0.005 |
| rs6549665 | 0.03 | 0.005 |
| rs658385 | 0.02 | 0.004 |
| rs726281 | 0.03 | 0.004 |
| rs76513770 | 0.03 | 0.006 |
| rs9516776 | 0.02 | 0.004 |
