# Supplementary material for: The effects of age at menarche and first sexual intercourse on reproductive and behavioural outcomes: A Mendelian randomization study
Source: PLoS One. 2020 Jun 15;15(6):e0234488. doi: 10.1371/journal.pone.0234488 (PMC7295202; doi:10.1371/journal.pone.0234488)
Supplement: S4 Table — (DOCX) [file pone.0234488.s007.docx]

**Table S4.** Estimates for the mean F statistic, I^2^_GX_, and Cochran’s Q.

|  | **Age at menarche** | | | | **Age at first sexual intercourse** | |
| --- | --- | --- | --- | --- | --- | --- |
|  | **116 SNPs** | | **305 SNPs** | |  |  |
| Mean F statistic | 60.98 | | 64.95 | | 39.22 | |
| Unweighted I^2^_GX_ | 0.9 | | 0.9 | | 0.6 | |
|  | **Q** | ***p*** | **Q** | ***p*** | **Q** | ***p*** |
| **Reproduction** |  |  |  |  |  |  |
| Age first birth | 323.47 | <0.001 | 575.69 | <0.001 | 52.01 | <0.001 |
| Age last birth | 273.22 | <0.001 | 402.69 | <0.001 | 41.96 | 0.01 |
| Reproductive period | 139.87 | 0.06 | 347.55 | 0.04 | 33.28 | 0.06 |
| Number of sexual partners | 178.63 | <0.001 | 466.96 | <0.001 | 23.87 | 0.35 |
| Number of children | 203.17 | <0.001 | 414.35 | <0.001 | 52.18 | <0.001 |
| Childlessness | 187.30 | <0.001 | 413.68 | <0.001 | 53.89 | <0.001 |
| **Education** |  |  |  |  |  |  |
| Age when left education | 233.24 | <0.001 | 442.82 | <0.001 | 33.27 | 0.06 |
| Educational attainment in years | 259.91 | <0.001 | 468.65 | <0.001 | 30.53 | 0.11 |
| **Risky behaviours** |  |  |  |  |  |  |
| Alcohol intake | 268.60 | <0.001 | 565.41 | <0.001 | 28.07 | 0.17 |
| Ever smoked | 227.40 | <0.001 | 561.59 | <0.001 | 67.12 | <0.001 |
| Risk taking | 146.57 | 0.03 | 392.27 | <0.001 | 38.48 | 0.02 |

Note: Cochran’s Q values indicate that most measures show evidence for over-dispersion, although the dispersion appeared balanced when plotted. Mean F statistic was calculated as the mean of the squared SNP-exposure association divided by the squared standard error for SNP-outcome association. The age at menarche 305 SNP instrument is using the non-overlapping UK Biobank sample.
