# Supplementary material for: The effects of age at menarche and first sexual intercourse on reproductive and behavioural outcomes: A Mendelian randomization study
Source: PLoS One. 2020 Jun 15;15(6):e0234488. doi: 10.1371/journal.pone.0234488 (PMC7295202; doi:10.1371/journal.pone.0234488)
Supplement: S5 Table — (DOCX) [file pone.0234488.s008.docx]

**Table S5**. Estimates of the causal effect of earlier age at menarche (116 SNPs) on life history outcomes using full UK Biobank data excluding SNPs associated with body mass index at *p*<5×10^-8^ (9 SNPs excluded).

|  |  | **IVW** | | **MR-Egger regression** | | **Weighted median** | | **MBE** | |
| --- | --- | --- | --- | --- | --- | --- | --- | --- | --- |
|  | **N** | **β or OR**  **(95% CI)** | ***p*** | **β or OR**  **(95% CI)** | ***p*** | **β or OR**  **(95% CI)** | ***p*** | **β or OR**  **(95% CI)** | ***p*** |
| **Reproduction** |  |  |  |  |  |  |  |  |  |
| Age first birth | 115070 - 124093 | -0.244  (-0.333, -0.155) | <0.001 | -0.215  (-0.479, 0.049) | 0.11 | -0.323  (-0.470, -0.176) | <0.001 | -0.356  (-0.703, -0.009) | 0.05 |
| Age last birth | 114916 - 123926 | -0.225  (-0.319, -0.130) | <0.001 | -0.188  (-0.469, 0.093) | 0.19 | -0.231  (-0.394, -0.068) | 0.01 | -0.205  (-0.500, 0.089) | 0.17 |
| Reproductive period | 114883 - 123892 | 0.015  (-0.058, 0.087) | 0.70 | 0.016  (-0.201, 0.232) | 0.89 | 0.013  (-0.100, 0.126) | 0.82 | -0.074  (-0.328, 0.180) | 0.57 |
| Number of sexual partners | 138920 - 149902 | -0.084  (-0.208, 0.039) | 0.18 | 0.101  (-0.266, 0.469) | 0.59 | -0.030  (-0.231, 0.170) | 0.77 | 0.016  (-0.397, 0.428) | 0.94 |
| Number of children | 168050 - 181247 | -0.019  (-0.038, -0.0003) | 0.05 | 0.016  (-0.040, 0.072) | 0.58 | -0.023  (-0.054, 0.008) | 0.15 | 0.018  (-0.044, 0.080) | 0.57 |
| Childlessness | 168058 - 181255 | 1.065  (1.020, 1.112) | 0.004 | 0.994  (0.874, 1.130) | 0.93 | 1.048  (0.978, 1.122) | 0.19 | 1.034  (0.898, 1.189) | 0.64 |
| **Education** |  |  |  |  |  |  |  |  |  |
| Age when left education | 115204 - 124279 | -0.061  (-0.101, -0.021) | 0.003 | -0.129  (-0.247, -0.011) | 0.03 | -0.102  (-0.165, -0.040) | 0.002 | -0.126  (-0.285, 0.033) | 0.12 |
| Educational attainment | 166640 - 179731 | -0.086  (-0.156, -0.016) | 0.02 | -0.242  (-0.451, -0.034) | 0.02 | -0.131  (-0.254, -0.009) | 0.04 | -0.232  (-0.475, 0.012) | 0.07 |
| **Risky behaviours** | | |  |  |  |  |  |  |  |
| Alcohol intake | 168039 - 181233 | 0.050  (0.025, 0.075) | <0.001 | -0.041  (-0.115, 0.034) | 0.28 | 0.020  (-0.022, 0.062) | 0.34 | 0.006  (-0.077, 0.089) | 0.88 |
| Ever smoked | 167584 - 180751 | 0.982  (0.950, 1.015) | 0.28 | 0.995  (0.901, 1.098) | 0.91 | 0.983  (0.931, 1.037) | 0.53 | 0.966  (0.864, 1.080) | 0.54 |
| Risk taking | 161994 - 174718 | 0.985  (0.943, 1.029) | 0.49 | 1.075  (0.944, 1.225) | 0.27 | 0.983  (0.911, 1.062) | 0.66 | 0.979  (0.835, 1.149) | 0.80 |

Note: Mendelian Randomization approaches used: inverse variance weighted, weighted mode-based estimator (MBE), MR-Egger regression and weighted median. (LCI: lower 95% confidence interval; UCI: upper 95% confidence interval; MBE: weighted mode-based estimator). The mean F statistics for this instrument was 61.07 and the I^2^_GX_ statistic was 0.9.
