# Supplementary material for: The effects of age at menarche and first sexual intercourse on reproductive and behavioural outcomes: A Mendelian randomization study
Source: PLoS One. 2020 Jun 15;15(6):e0234488. doi: 10.1371/journal.pone.0234488 (PMC7295202; doi:10.1371/journal.pone.0234488)
Supplement: S6 Table — (DOCX) [file pone.0234488.s009.docx]

**Table S6.** MR-Egger intercept values for age at menarche (116 SNPs) on life history outcomes using full UK Biobank data and excluding SNPs associated with body mass index at *p*<5×10^-8^ (9 SNPs excluded).

|  | **MR-Egger intercept** | | | |
| --- | --- | --- | --- | --- |
|  | **β or OR** | **95% CI** | | ***p*** |
| **Reproduction** |  |  |  |  |
| Age first birth | -0.001 | -0.013, 0.010 | | 0.82 |
| Age last birth | -0.002 | -0.014, 0.011 | | 0.78 |
| Reproductive period | -0.0001 | -0.010, 0.010 | | 0.99 |
| Number of sexual partners | -0.009 | -0.025, 0.008 | | 0.29 |
| Number of children | -0.002 | -0.004, 0.001 | | 0.19 |
| Childlessness | 1.003 | 0.998, 1.009 | | 0.26 |
| **Education** |  |  |  |  |
| Age when left education | 0.003 | -0.002, 0.008 | | 0.23 |
| Educational attainment | 0.007 | -0.002, 0.016 | | 0.12 |
| **Risky behaviours** |  |  |  |  |
| Alcohol intake | 0.004 | 0.001, 0.008 | | 0.01 |
| Ever smoked | 0.999 | 0.995, 1.004 | | 0.79 |
| Risk taking | 0.996 | 0.990, 1.002 | | 0.16 |

Note: LCI: lower 95% confidence interval; UCI: upper 95% confidence interval.
