# Supplementary material for: The effects of age at menarche and first sexual intercourse on reproductive and behavioural outcomes: A Mendelian randomization study
Source: PLoS One. 2020 Jun 15;15(6):e0234488. doi: 10.1371/journal.pone.0234488 (PMC7295202; doi:10.1371/journal.pone.0234488)
Supplement: S7 Table — (DOCX) [file pone.0234488.s010.docx]

**Table S7.** Estimates of the causal effect of earlier age at menarche (305 SNPs) on life history outcomes using non-overlapping UK Biobank data.

|  |  | **IVW** | | **MR-Egger regression** | | **Weighted Median** | | **MBE** | |
| --- | --- | --- | --- | --- | --- | --- | --- | --- | --- |
|  | **N** | **β or OR**  **(95% CI)** | ***p*** | **β or OR**  **(95% CI)** | ***p*** | **β or OR**  **(95% CI)** | ***p*** | **β or OR**  **(95% CI)** | ***p*** |
| **Reproduction** | | | | | | | | | |
| Age first birth | 75469 - 90503 | -0.286  (-0.361, -0.211) | <0.001 | -0.342  (-0.540, -0.144) | 0.001 | -0.320  (-0.444, -0.195) | <0.001 | -0.261  (-0.607, 0.086) | 0.14 |
| Age last birth | 75366 - 90382 | -0.255  (-0.335, -0.175) | <0.001 | -0.224  (-0.435, -0.013) | 0.04 | -0.174  (-0.307, -0.041) | 0.01 | -0.119  (-0.434, 0.196) | 0.46 |
| Reproductive period | 75343 - 90357 | 0.027  (-0.035, 0.089) | 0.39 | 0.115  (-0.047, 0.278) | 0.16 | 0.030  (-0.076, 0.136) | 0.58 | 0.055  (-0.223, 0.332) | 0.70 |
| Number of sexual partners | 90768 - 108801 | -0.116  (-0.218, -0.015) | 0.03 | 0.110  (-0.068, 0.467) | 0.14 | 0.027  (-0.152, 0.206) | 0.77 | 0.113  (-0.289, 0.515) | 0.58 |
| Number of children | 109636 - 131506 | -0.002  (-0.018, 0.014) | 0.77 | 0.015  (-0.027, 0.057) | 0.49 | -0.003  (-0.029, 0.023) | 0.80 | 0.067  (-0.019, 0.154) | 0.13 |
| Childlessness | 109641 - 131512 | 1.036  (0.998, 1.075) | 0.06 | 1.074  (0.974, 1.184) | 0.15 | 1.028  (0.968, 1.091) | 0.38 | 0.982  (0.841, 1.146) | 0.82 |
| **Education** |  |  |  |  |  |  |  |  |  |
| Age when left education | 75041 - 89959 | -0.051  (-0.085, -0.017) | 0.003 | -0.012  (-0.101, 0.077) | 0.80 | -0.074  (-0.130, -0.018) | 0.01 | 0.049  (-0.143, 0.241) | 0.62 |
| Educational attainment | 108704 - 130387 | -0.088  (-0.148, -0.029) | 0.004 | -0.207  (-0.364, -0.049) | 0.01 | -0.211  (-0.311, -0.111) | <0.001 | -0.283  (-0.529, -0.037) | 0.03 |
| **Risky behaviours** |  |  |  |  |  |  |  |  |  |
| Alcohol intake | 109615 - 131487 | 0.051  (0.030, 0.073) | <0.001 | 0.037  (-0.019, 0.092) | 0.20 | 0.040  (0.003, 0.077) | 0.04 | 0.011  (-0.086, 0.107) | 0.83 |
| Ever smoked | 109288 - 131109 | 0.970  (0.943, 0.998) | 0.04 | 1.040  (0.964, 1.121) | 0.31 | 0.963  (0.914, 1.014) | 0.16 | 0.968  (0.861, 1.089) | 0.59 |
| Risk taking | 105717 - 126762 | 0.991  (0.955, 1.029) | 0.65 | 1.057  (0.958, 1.166) | 0.27 | 0.992  (0.929, 1.058) | 0.80 | 1.035  (0.877, 1.222) | 0.68 |

Note: Mendelian Randomization approaches used: inverse variance weighted, weighted mode-based estimator (MBE), MR-Egger regression and weighted median. (LCI: lower 95% confidence interval; UCI: upper 95% confidence interval; MBE: weighted mode-based estimator).
