# Supplementary material for: The effects of age at menarche and first sexual intercourse on reproductive and behavioural outcomes: A Mendelian randomization study
Source: PLoS One. 2020 Jun 15;15(6):e0234488. doi: 10.1371/journal.pone.0234488 (PMC7295202; doi:10.1371/journal.pone.0234488)
Supplement: S8 Table — (DOCX) [file pone.0234488.s011.docx]

**Table S8.** MR-Egger intercept values for age at menarche (305 SNPs) on life history outcomes using non-overlapping UK Biobank data.

|  | **MR-Egger intercept** | | | |
| --- | --- | --- | --- | --- |
|  | **β or OR** | **95% CI** | | ***p*** |
| **Reproduction** |  |  |  |  |
| Age first birth | 0.002 | -0.005, 0.010 | | 0.55 |
| Age last birth | -0.001 | -0.009, 0.007 | | 0.76 |
| Reproductive period | -0.004 | -0.010, 0.003 | | 0.25 |
| Number of sexual partners | -0.013 | -0.023, -0.003 | | 0.01 |
| Number of children | -0.001 | -0.002, 0.001 | | 0.39 |
| Childlessness | 0.999 | 0.995, 1.002 | | 0.44 |
| **Education** |  |  |  |  |
| Age when left education | -0.002 | -0.005, 0.002 | | 0.35 |
| Educational attainment | 0.005 | -0.001, 0.011 | | 0.11 |
| **Risky behaviours** |  |  |  |  |
| Alcohol intake | 0.001 | -0.002, 0.003 | | 0.57 |
| Ever smoked | 0.997 | 0.994, 1.000 | | 0.05 |
| Risk taking | 0.997 | 0.994, 1.001 | | 0.17 |

Note: LCI: lower 95% confidence interval; UCI: upper 95% confidence interval.
