# Supplementary material for: The effects of age at menarche and first sexual intercourse on reproductive and behavioural outcomes: A Mendelian randomization study
Source: PLoS One. 2020 Jun 15;15(6):e0234488. doi: 10.1371/journal.pone.0234488 (PMC7295202; doi:10.1371/journal.pone.0234488)
Supplement: S9 Table — (DOCX) [file pone.0234488.s012.docx]

**Table S9.** Estimates of the causal effect of earlier age at menarche (305 SNPs) on life history outcomes using full UK Biobank data.

|  |  | **IVW** | | **MR-Egger regression** | | **Weighted median** | | **MBE** | |
| --- | --- | --- | --- | --- | --- | --- | --- | --- | --- |
|  | **N** | **β or OR**  **(95% CI )** | ***p*** | **β or OR**  **(95% CI )** | ***p*** | **β or OR**  **(95% CI )** | ***p*** | **β or OR**  **(95% CI )** | ***p*** |
| **Reproduction** |  |  |  |  |  |  |  |  |  |
| Age first birth | 103472 - 124093 | -0.325  (-0.390, -0.261) | <0.001 | -0.263  (-0.433, -0.093) | 0.003 | -0.314  (-0.423, -0.205) | <0.001 | -0.290  (-0.625, 0.044) | 0.09 |
| Age last birth | 103332 - 123926 | -0.287  (-0.356, -0.219) | <0.001 | -0.176  (-0.357, 0.005) | 0.06 | -0.236  (-0.357, -0.115) | <0.001 | -0.116  (-0.406, 0.175) | 0.44 |
| Reproductive period | 103302 - 123892 | 0.034  (-0.019, 0.087) | 0.21 | 0.084  (-0.056, 0.223) | 0.24 | 0.025  (-0.063, 0.112) | 0.58 | 0.034  (-0.232, 0.301) | 0.80 |
| Number of sexual partners | 125058 - 149902 | -0.089  (-0.179, 0.001) | 0.05 | 0.006  (-0.231, 0.242) | 0.96 | -0.049  (-0.217, 0.118) | 0.56 | 0.010  (-0.344, 0.363) | 0.96 |
| Number of children | 151104 - 181247 | -0.004  (-0.018, 0.010) | 0.58 | -0.010  (-0.045, 0.026) | 0.60 | -0.009  (-0.032, 0.015) | 0.46 | 0.002  (-0.055, 0.060) | 0.94 |
| Childlessness | 151111 - 181255 | 1.032  (1.000, 1.065) | 0.05 | 1.096  (1.009, 1.190) | 0.03 | 1.056  (1.001, 1.114) | 0.05 | 1.076  (0.944, 1.227) | 0.27 |
| **Education** |  |  |  |  |  |  |  |  |  |
| Age when left education | 103643 - 124267 | -0.060  (-0.089, -0.031) | <0.001 | -0.065  (-0.141, 0.012) | 0.10 | -0.068  (-0.115, -0.020) | 0.01 | -0.022  (-0.185, 0.141) | 0.79 |
| Educational attainment | 149833 - 179731 | -0.048  (-0.099, 0.003) | 0.06 | -0.148  (-0.282, -0.013) | 0.03 | -0.126  (-0.220, -0.032) | 0.01 | -0.154  (-0.386, 0.077) | 0.19 |
| **Risky behaviours** | |  |  |  |  |  |  |  |  |
| Alcohol intake | 151085 - 181233 | 0.052  (0.034, 0.070) | <0.001 | 0.050  (0.002, 0.097) | 0.04 | 0.050  (0.018, 0.083) | 0.002 | 0.010  (-0.080, 0.101) | 0.82 |
| Ever smoked | 150677 - 180751 | 0.972  (0.949, 0.996) | 0.02 | 1.027  (0.962, 1.094) | 0.43 | 0.971  (0.929, 1.014) | 0.19 | 0.976  (0.883, 1.078) | 0.63 |
| Risk taking | 145713 - 174718 | 0.999  (0.967, 1.031) | 0.94 | 1.080  (0.993, 1.175) | 0.07 | 0.989  (0.933, 1.048) | 0.72 | 1.000  (0.855, 1.171) | 0.10 |

Note: Mendelian Randomization approaches used: inverse variance weighted, weighted mode-based estimator (MBE), MR-Egger regression and weighted median. (LCI: lower 95% confidence interval; UCI: upper 95% confidence interval; MBE: weighted mode-based estimator).
