# Supplementary material for: The effects of age at menarche and first sexual intercourse on reproductive and behavioural outcomes: A Mendelian randomization study
Source: PLoS One. 2020 Jun 15;15(6):e0234488. doi: 10.1371/journal.pone.0234488 (PMC7295202; doi:10.1371/journal.pone.0234488)
Supplement: S10 Table — (DOCX) [file pone.0234488.s013.docx]

**Table S10.** MR-Egger intercept values for age at menarche (305 SNPs) on life history outcomes using full UK Biobank data.

|  | **MR-Egger intercept** | | | |
| --- | --- | --- | --- | --- |
|  | **β or OR** | **95% CI** | | ***p*** |
| **Reproduction** |  |  |  |  |
| Age first birth | -0.003 | -0.009, 0.004 | | 0.44 |
| Age last birth | -0.005 | -0.011, 0.002 | | 0.19 |
| Reproductive period | -0.002 | -0.007, 0.003 | | 0.45 |
| Number of sexual partners | -0.004 | -0.013, 0.005 | | 0.40 |
| Number of children | 0.0002 | -0.001, 0.002 | | 0.74 |
| Childlessness | 0.998 | 0.994, 1.001 | | 0.12 |
| **Education** |  |  |  |  |
| Age when left education | 0.0002 | -0.003, 0.003 | | 0.90 |
| Educational attainment | 0.004 | -0.001, 0.009 | | 0.12 |
| **Risky behaviours** |  |  |  |  |
| Alcohol intake | 0.0001 | -0.002, 0.002 | | 0.92 |
| Ever smoked | 0.998 | 0.995, 1.000 | | 0.07 |
| Risk taking | 0.997 | 0.994, 1.000 | | 0.05 |

Note: LCI: lower 95% confidence interval; UCI: upper 95% confidence interval.
