# Supplementary material for: The effects of age at menarche and first sexual intercourse on reproductive and behavioural outcomes: A Mendelian randomization study
Source: PLoS One. 2020 Jun 15;15(6):e0234488. doi: 10.1371/journal.pone.0234488 (PMC7295202; doi:10.1371/journal.pone.0234488)
Supplement: S11 Table — (DOCX) [file pone.0234488.s014.docx]

**Table S11.** Fixed effects meta-analysis of SNP-outcome associations using full UK Biobank and SNPs identified for age at first sexual intercourse (23 SNPs).

|  |  | **Fixed effects meta-analysis** | | |
| --- | --- | --- | --- | --- |
|  | **N** | **β or OR** | **95% CI** | ***p*** |
| **Reproduction** | | | | |
| Age first birth | 109 021 – 124 093 | -0.061 | -0.069, -0.053 | <0.001 |
| Age last birth | 108 873 – 123 926 | -0.046 | -0.055, -0.037 | <0.001 |
| Reproductive period | 108 842 – 123 892 | 0.015 | 0.008, 0.022 | <0.001 |
| Number of sexual partners | 131 643 – 149 902 | 0.019 | 0.007, 0.030 | 0.002 |
| Number of children | 159 140 – 181 247 | 0.006 | 0.004, 0.008 | <0.001 |
| Childlessness | 159 147 – 181 255 | 0.986 | 0.982, 0.990 | <0.001 |
| **Education** | | | | |
| Age when left education | 109 137 – 124 279 | -0.011 | -0.015, -0.007 | <0.001 |
| Educational attainment in years | 157 817 – 179 731 | -0.015 | -0.022, -0.008 | <0.001 |
| **Risky** **behaviours** | | | | |
| Alcohol intake | 159 137 – 181 233 | 0.001 | -0.001, 0.003 | 0.409 |
| Ever smoked | 158 702 – 180 751 | 1.010 | 1.007, 1.014 | <0.001 |
| Risk taking | 153 432 – 174 718 | 1.011 | 1.006, 1.015 | <0.001 |

Note: LCI: lower 95% confidence interval; UCI: upper 95% confidence interval.
