# Supplementary material for: The effects of age at menarche and first sexual intercourse on reproductive and behavioural outcomes: A Mendelian randomization study
Source: PLoS One. 2020 Jun 15;15(6):e0234488. doi: 10.1371/journal.pone.0234488 (PMC7295202; doi:10.1371/journal.pone.0234488)
Supplement: S12 Table — (DOCX) [file pone.0234488.s015.docx]

**Table S12.** Estimates of the causal effect of earlier age at first sexual intercourse on life history outcomes using non-overlapping UK Biobank data.

|  |  | **IVW** | | **SIMEX MR-Egger regression** | | **Weighted median** | | **MBE** | |
| --- | --- | --- | --- | --- | --- | --- | --- | --- | --- |
|  | **N** | **β or OR**  **(95% CI)** | ***p*** | **β or OR**  **(95% CI)** | ***p*** | **β or OR**  **(95% CI)** | ***p*** | **β or OR**  **(95% CI)** | ***p*** |
| **Reproduction** | |  |  |  |  |  |  |  |  |
| Age first birth | 79494 - 90503 | -2.146  (-2.524, -1.768) | <0.001 | 0.738  (0.480, 0.996) | <0.001 | -2.301  (-2.864, -1.739) | <0.001 | -2.535  (-3.858, -1.211) | 0.001 |
| Age last birth | 79387 - 90382 | -1.687  (-2.090, -1.284) | <0.001 | -0.611  (-0.883, -0.339) | <0.001 | -1.546  (-2.143, -0.950) | <0.001 | -1.567  (-2.855, -0.278) | 0.03 |
| Reproductive period | 79365 - 90357 | 0.456  (0.146, 0.767) | 0.01 | -1.371  (-1.625, -1.118) | <0.001 | 0.341  (-0.086, 0.768) | 0.13 | 0.403  (-0.569, 1.375) | 0.43 |
| Number of sexual partners | 95510 - 108801 | 0.327  (-0.184, 0.838) | 0.20 | 0.039  (-0.192, 0.269) | 0.75 | -0.155  (-0.828, 0.517) | 0.66 | -0.184  (-1.668, 1.301) | 0.81 |
| Number of children | 115445 - 131506 | 0.223  (0.143, 0.303) | <0.001 | -0.167  (-0.247, -0.086) | 0.001 | 0.191  (0.067, 0.316) | 0.006 | 0.044  (-0.276, 0.363) | 0.79 |
| Childlessness | 115450 - 131512 | 0.605  (0.502, 0.728) | <0.001 | 1.021  (0.908, 1.147) | 0.74 | 0.732  (0.557, 0.963) | 0.04 | 0.905  (0.504, 1.625) | 0.74 |
| **Education** |  |  |  |  |  |  |  |  |  |
| Age when left education | 78953 - 89959 | -0.285  (-0.456, -0.115) | 0.002 | 0.596  (0.357, 0.835) | <0.001 | -0.160  (-0.401, 0.081) | 0.21 | -0.112  (-0.648, 0.424) | 0.69 |
| Educational attainment | 114477 - 130387 | -0.484  (-0.784, -0.183) | 0.003 | 0.109  (-0.036, 0.255) | 0.15 | -0.247  (-0.659, 0.164) | 0.25 | 0.045  (-0.818, 0.907) | 0.92 |
| **Risky behaviours** | |  |  |  |  |  |  |  |  |
| Alcohol intake | 115436 - 131487 | 0.026  (-0.081, 0.133) | 0.62 | -0.375  (-0.498, -0.252) | <0.001 | 0.049  (-0.102, 0.200) | 0.53 | 0.077  (-0.256, 0.410) | 0.66 |
| Ever smoked | 115090 - 131109 | 1.393  (1.206, 1.608) | <0.001 | 0.567  (0.492, 0.653) | <0.001 | 1.391  (1.103, 1.754) | 0.01 | 1.425  (0.751, 2.704) | 0.29 |
| Risk taking | 111292 - 126762 | 1.348  (1.117, 1.626) | 0.003 | 1.603  (1.297, 1.982) | <0.001 | 1.527  (1.177, 1.982) | 0.004 | 1.523  (0.813, 2.851) | 0.20 |

Note: Mendelian Randomization approaches used: inverse variance weighted, weighted mode-based estimator (MBE), MR-Egger regression and weighted median. (LCI: lower 95% confidence interval; UCI: upper 95% confidence interval; MBE: weighted mode-based estimator).
