# Supplementary material for: The effects of age at menarche and first sexual intercourse on reproductive and behavioural outcomes: A Mendelian randomization study
Source: PLoS One. 2020 Jun 15;15(6):e0234488. doi: 10.1371/journal.pone.0234488 (PMC7295202; doi:10.1371/journal.pone.0234488)
Supplement: S13 Table — (DOCX) [file pone.0234488.s016.docx]

**Table S13.** SIMEX unweighted MR-Egger intercept values for age at first sexual intercourse on life history outcomes using non-overlapping UK Biobank data.

|  | **MR-Egger intercept** | | | | |
| --- | --- | --- | --- | --- | --- |
|  | **β or OR** | **95% CI** | |  | ***p*** |
| **Reproduction** |  |  |  |  |  |
| Age first birth | -0.082 | -0.101, -0.063 | |  | <0.001 |
| Age last birth | -0.030 | -0.048, -0.013 | |  | 0.002 |
| Reproductive period | 0.052 | 0.036, 0.069 | |  | <0.001 |
| Number of sexual partners | 0.009 | -0.008, 0.027 | |  | 0.30 |
| Number of children | 0.011 | 0.006, 0.015 | |  | <0.001 |
| Childlessness | 0.986 | 0.977, 0.995 | |  | 0.01 |
| **Education** |  |  |  |  |  |
| Age when left education | -0.025 | -0.034, -0.016 | |  | <0.001 |
| Educational attainment in years | -0.017 | -0.027, -0.006 | |  | 0.01 |
| **Risky behaviours** |  |  |  |  |  |
| Alcohol intake | 0.011 | 0.006, 0.017 | |  | 0.001 |
| Ever smoked | 1.026 | 1.018, 1.035 | |  | <0.001 |
| Risk taking | 0.995 | 0.985, 1.004 | |  | 0.27 |

Note: LCI: lower 95% confidence interval; UCI: upper 95% confidence interval.
